# Alcohol-associated hepatitis: a neutrophile disease?

**DOI:** 10.1136/gutjnl-2024-333222

**Published:** 2024-09-24

**Authors:** Maximilian Joseph Brol, Ali Canbay, Jonel Trebicka

**Affiliations:** 1 Department of Internal Medicine B, University of Münster, Munster, Nordrhein-Westfalen, Germany; 2 Department of Internal Medicine, Ruhr-Universitat Bochum, Bochum, Germany; 3 European Foundation for the Study of Chronic Liver Failure, Barcelona, Spain

**Keywords:** ALCOHOL, ALCOHOLIC LIVER DISEASE, HEPATITIS, GUT IMMUNOLOGY, LIVER IMMUNOLOGY

 Alcohol-associated hepatitis (AH) is the acute deterioration of alcohol-related liver disease (ArLD) with rapid onset or worsening of jaundice, which, in severe cases, may transition to acute-on-chronic liver failure (ACLF) with extremely high short-term mortality, increasing with the number and severity of hepatic and extra-hepatic organ dysfunction. Systemic inflammation is a hallmark, driving acute decompensation (AD) towards ACLF. Diagnosis and treatment are insufficient and challenging, especially due to the complex, multifactorial and as yet not fully understood pathogenesis. In patients with AH, this inflammation is characterised by increased levels of circulating and hepatic neutrophils, which are essential immune cells responsible for pathogen defence. However, the exact role of neutrophils in AH remains controversial, with ongoing debate over whether their hyperactivation exacerbates liver damage or helps to resolve the disease.

Current treatment for AH primarily relies on steroids, but their use is restricted in cases of bacterial infections. Consequently, there is a clinical need to better understand the mechanisms underlying AH and the associated organ dysfunction. Moreover, early detection and treatment of bacterial infections are critical to improve patient outcomes. These challenges, coupled with its rising prevalence in Germany and other Western countries, highlight a significant gap in patient care.^1^

Chronic alcohol consumption is associated with gut dysbiosis, leading to alterations in the composition of bacteria, viruses and fungi. Several bacterial metabolites were identified to foster liver disease progression. Among them, cytolysin, an endotoxin secreted by *Enterococcus faecalis,* is associated with higher hepatic inflammation and higher short-term mortality in patients with AH.^2^ Interestingly, this is not the case for patients with late-stage alcohol-associated liver disease, where faecal cytolysin was not able to predict the onset of ACLF in patients with AD.^3^

Nonetheless, such changes contribute to liver damage and provoke intestinal inflammation by activating macrophages and monocytes, which in turn disrupt the epithelial barrier. This disruption permits the translocation of microbial-associated molecular patterns (MAMPs), intensifying systemic immune responses and liver inflammation. Recent data indicate that with increasing liver disease severity, tight junction proteins like villin-1 are less expressed in the small bowel and can be detected in serum. Their levels correlate with mortality, proposing intestinal failure as additional organ failure to predict short-term mortality in ACLF.^4^ Further identification of key molecules is crucial for a better understanding of the mechanisms in AH and is urgently needed for patients’ stratification.

It is therefore of particular interest that Kreimeyer *et al* focused on analysing the faecal proteome in patients with ArLD, specifically comparing those with AH to individuals with alcohol use disorder and healthy controls. A distinct proteomic difference was found between healthy individuals and those with ArLD, with the most pronounced changes being the upregulation of proteins related to neutrophil function and degranulation.^5^ Myeloperoxidase (MPO), a marker of azurophil granules, was significantly elevated in the faecal samples of patients with AH. This increase not only correlated with the severity of the disease but was also linked to a higher 60-day mortality rate, rendering this protein highly clinically relevant.

A recent study identified orosomucoid 2 (ORM2) as a key regulatory protein in de novo lipogenesis in non-alcoholic steatohepatitis and showed preclinical data on beneficial pharmacological targeting.^6^ In addition, Kreimeyer *et al* demonstrated faecal ORM2 to be significantly associated with AH and showed its increase in faecal samples dependent on the disease severity. Given the fact hepatic steatosis is a histological hallmark of AH, this might be a common mechanistic pathway between AH and metabolic liver diseases.

**Figure 1:**
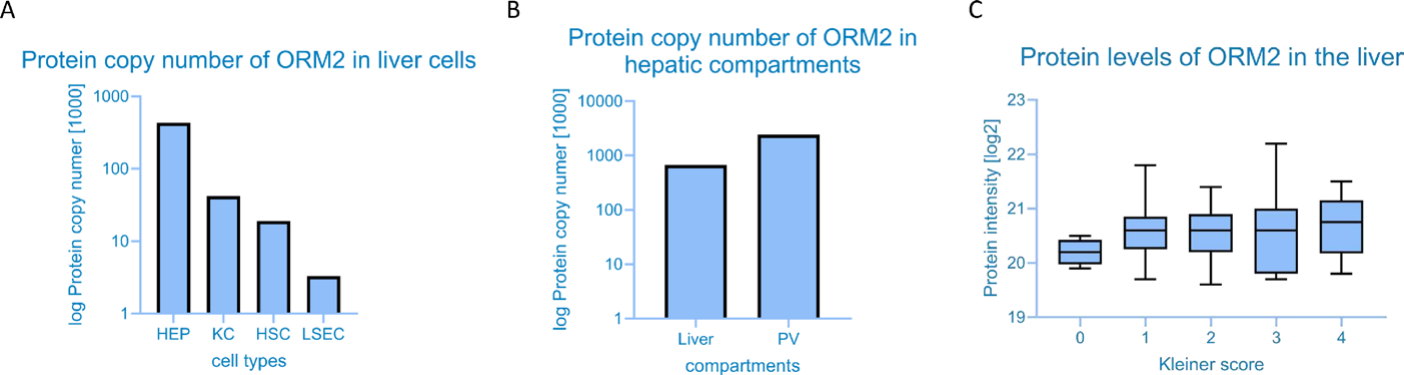
Abundance and role of ORM2 in ArLD. (**A**) Protein copy number of ORM2 across four major liver cell types: hepatocytes, Kupffer cells, hepatic stellate cells and liver sinusoidal endothelial cells. (**B**) Protein level of ORM2 in the liver and in the portal vein. For both, data are extracted from http://www.liverproteome.org/, the largest cell-type resolved human liver proteome data by MS-based proteomics hosting quantitative information about proteins across four major liver cell types: hepatocytes, sinusoidal endothelial cells, hepatic stellate cells and Kupffer cells.^9^ (**C**) Protein level of ORM2 in the liver of patients with ArLD classified by fibrosis according to Kleiner fibrosis score. Data are extracted from proteome data of patients with ArLD, presenting dysregulated proteome at both protein and pathway levels in liver and plasma, publicly available at http://www.liverproteome.org.^10^ ArLD, alcohol-related liver disease; ORM2, orosomucoid 2.

**Figure 2:**
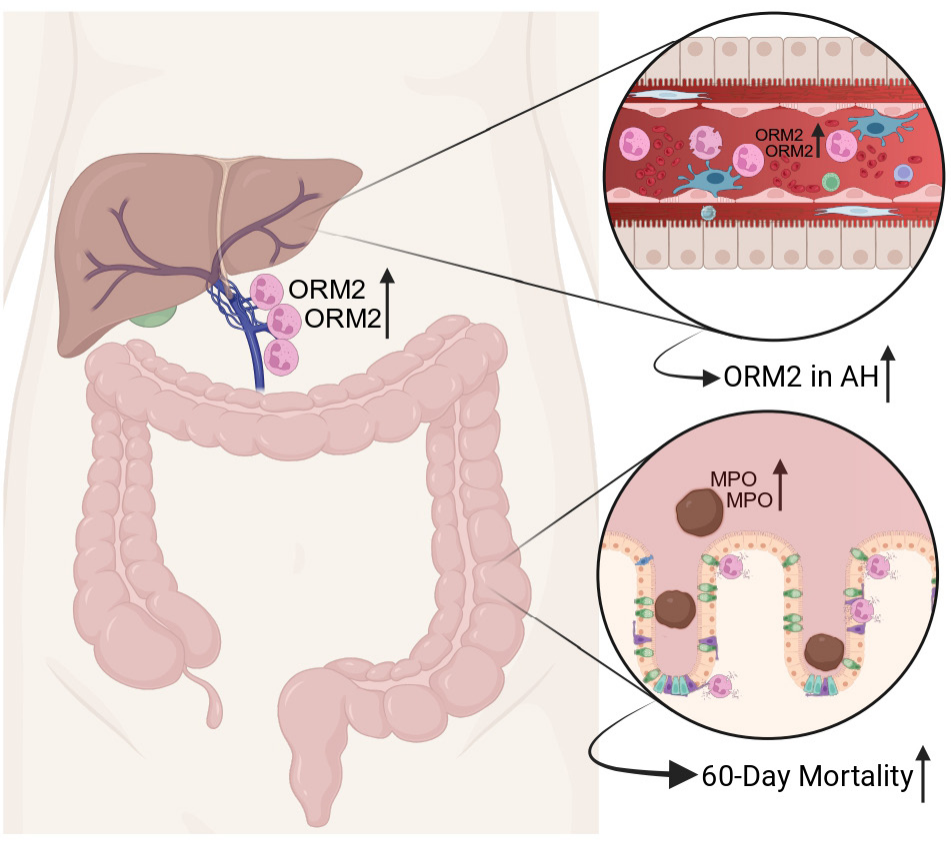
Schematic overview of the gut-liver axis in alcohol-associated hepatitis. Faecal MPO is increased in alcoholic hepatitis and associated with an increased 60-day mortality. ORM2 is expressed in neutrophils and is highly abundant in the portal vein. As the severity of alcoholic hepatitis increases, hepatic ORM2 levels are rising (created with BioRender.com). AH, alcohol-associated hepatitis; MPO, myeloperoxidase; ORM2, orosomucoid 2.

Indeed, ORM2 is primarily expressed in hepatocytes, but it can also be detected in other cell types in the liver, mainly Kupffer cells, as we can show by in silico analysis of human proteome data (figure 1A). The close relationship between Kupffer cells and hepatic neutrophil infiltration is already well established.^7^ Bearing in mind the important immunological function of hepatocytes, ORM2 seems to be linked to immunological functions in the liver. When looking into its abundance in hepatic compartments, the highest values were observed in the portal vein, underlining its particular role in the gut-liver axis (figure 1B). In analogy to Kreimeyer *et al*, hepatic ORM2 shows increasing values with increasing disease severity in ArLD (figure 1C). Moreover, ORM2 is further highly expressed in neutrophils,^8^ implicating their important role in ArLD. However, little is known yet about its pathophysiological contribution. Taken together, Kreimeyer *et al* draw attention to ORM2, which seems to play an important role across all stages of alcohol-associated liver disease. These findings are visualised in figure 2.

In summary, the work of Kreimeyer *et al* identified faecal proteins, which play an important role in the pathophysiology of alcoholic hepatitis. This enlarged our perspective to study gut-liver crosstalk in ArLD and strongly encouraged further research on faecal proteomics and gut dysbiosis.
